# Supplementary material for: Hepatic metabolic reprogramming in male mice during short-term caloric restriction involves enhanced glucocorticoid rhythms
Source: Nat Commun. 2025 Dec 11;16:11106. doi: 10.1038/s41467-025-67228-z (PMC12700935; doi:10.1038/s41467-025-67228-z)
Supplement: Supplementary file 1 — Supplementary Information [file 41467_2025_67228_MOESM1_ESM.pdf]

Supplementary Information

Hepatic metabolic reprogramming in male mice during short-term caloric restriction involves enhanced glucocorticoid rhythms

Makris et al.

Suppl. Fig. 1

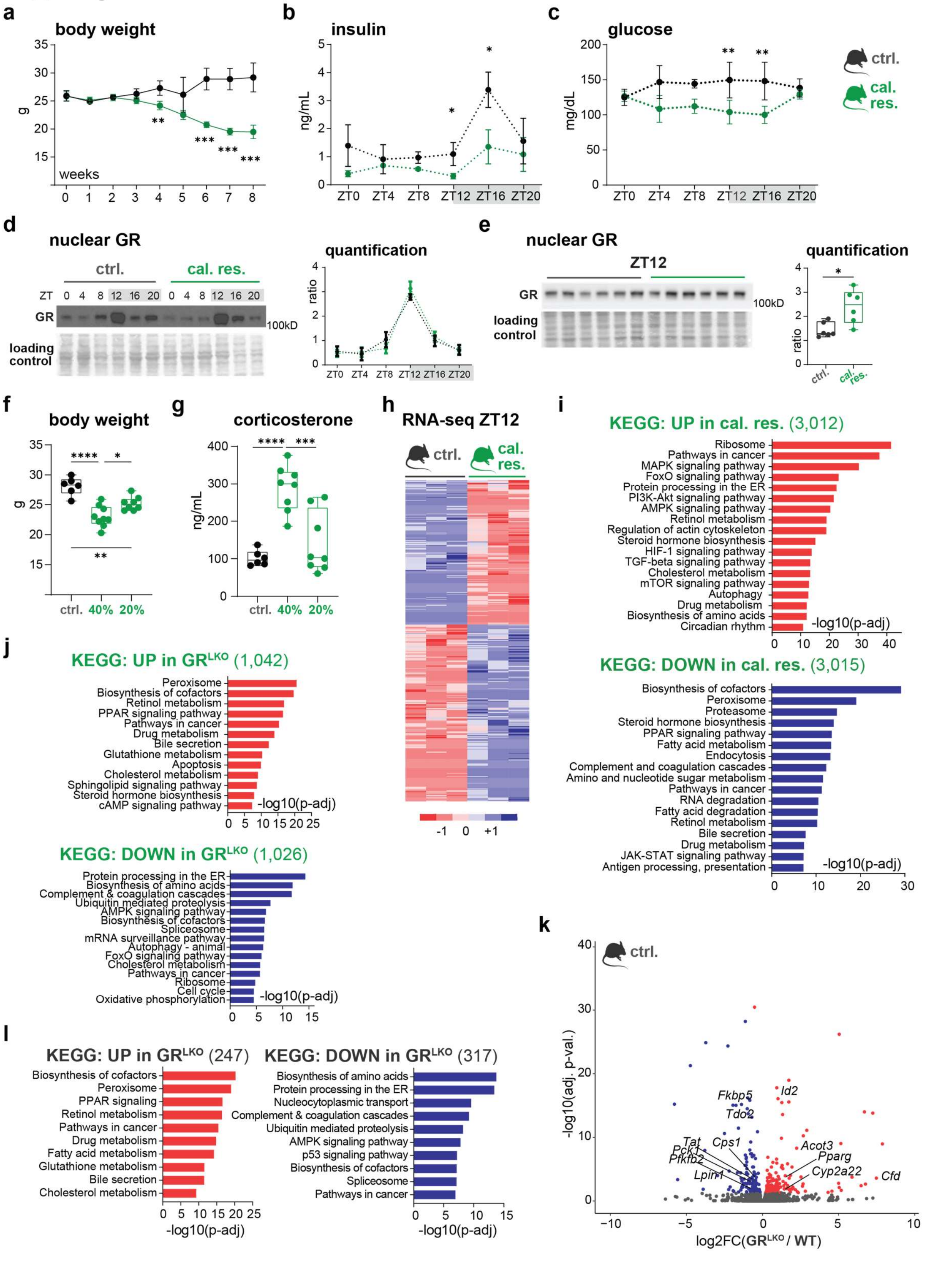

Supplementary Fig. 1, related to Fig. 1

a, Body weight trajectories of representative control-fed (ctrl.) and caloric restriction (cal. res.) mice (n=6). Mean ± SD measured weekly for 8 weeks. Stats: repeated measures ANOVA with Šídák post-hoc correction. **b-c**, Circulating insulin (b; n=3), and glucose (c; n=4-8), in WT mice under ctrl. and cal. res. diet sampled every 4 h at the end of the feeding regimen. Group comparisons at ZT12 and ZT16 by unpaired t-test. **d**, Representative western blots and quantifications (immunoblot signal normalized to

the loading control) for nuclear GR in ctrl. and cal. res. wildtype livers collected every 4 hours (n=3). **e**, Independent replicate samples highlighting the ZT12 difference (n=6), two-tailed unpaired t-test. **f-g**, Body weight (f) and corticosterone levels (g) after 8 weeks of 40% or 20% cal. res. Mice fed *ad libitum* during the dark phase were used as controls (ctrl.) (n=6-8). Biological replicates were collected at ZT12 (6 p.m.). Box plots display median (central line), 25th-75th percentiles (box), and min-max whiskers, with individual points shown. Statistical significance was assessed by one-way ANOVA followed by post-hoc tests adjusted for multiple comparisons. **h**, Bulk RNA-seq heatmap (z-scores) displaying significantly up- and down-regulated transcripts in WT ctrl. and WT cal. res. Analysis was performed by DESeq2 (Wald test, B-H FDR). Genes with FDR <0.05 are shown (ZT12, n=3). **i**, KEGG pathway annotation for up- and down-regulated genes in WT under cal. res. vs. ctrl. **j**, KEGG pathway annotation for up- and down-regulated genes in hepatocyte-specific GR knockouts, GR^{LKO} vs. WT under caloric restriction. **k**, Volcano plot of transcripts differentially regulated in GR^{LKO} compared to WT under ctrl. diet (DESeq2; Wald test, B-H FDR). Genes with p-adj <0.05 are highlighted (n=3-4). Red indicates up-regulated, and blue indicates down-regulated genes. **l**, KEGG pathway annotation for up- and down-regulated genes in GR^{LKO} vs. WT under ctrl. diet. g:Profiler (Gene Ontology Statistics) enrichment; p-values adjusted by B-H FDR. Source data and exact n per condition are provided in the Source Data file. *p<0.05, **p<0.01, ***p<0.001, and ****p<0.0001.

Suppl. Fig. 2

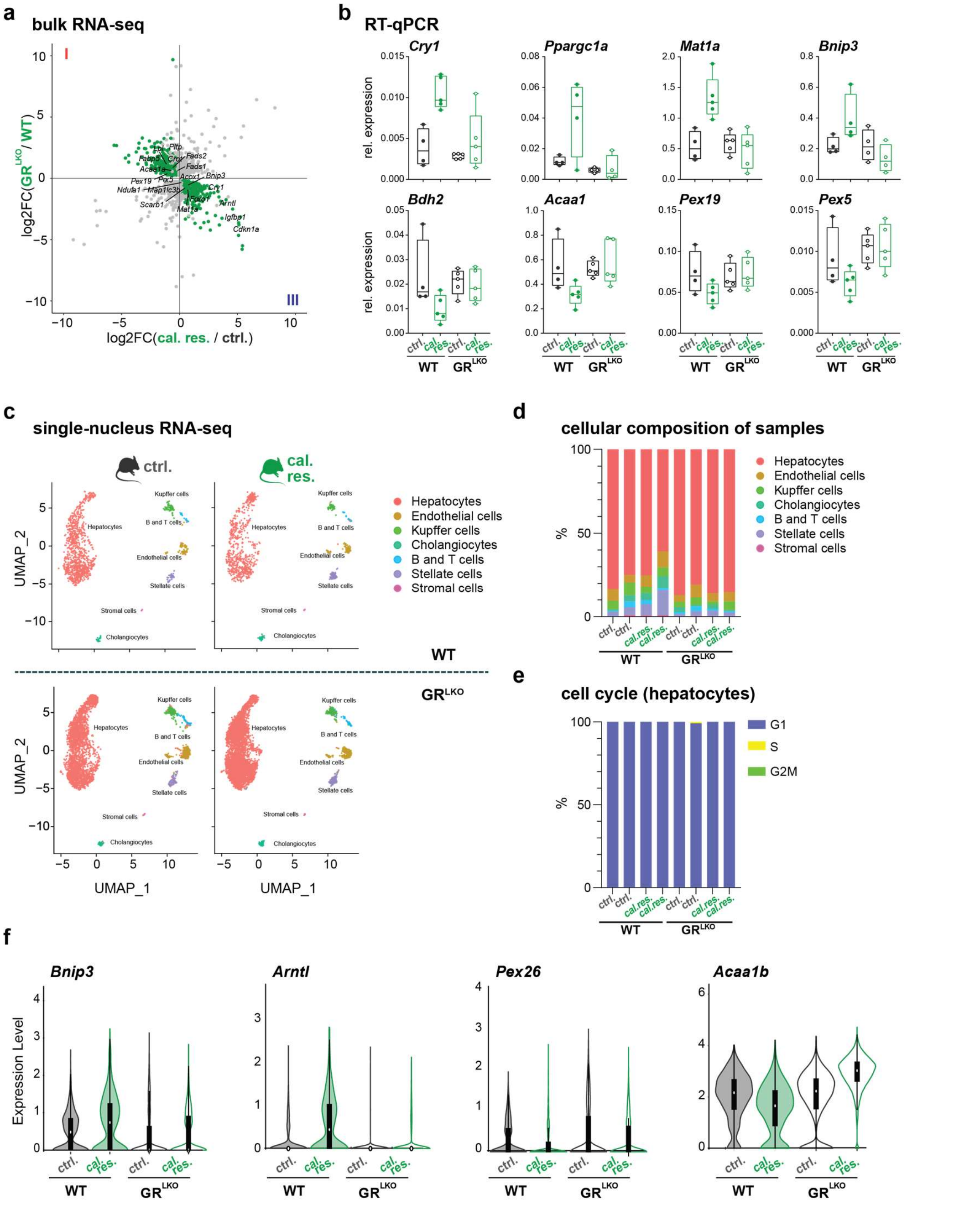

Supplementary Fig. 2, related to Fig. 1

a, Quadrant plot comparing fold changes in gene expression between 'WT cal. res. vs WT ctrl.' and 'GR^{LKO} cal. res. vs WT cal. res.'. Transcripts regulated in opposite directions in the absence of GR are shown in green (quadrant I: Down in cal. res. but Up in GR^{LKO}; quadrant III: Up in cal. res. but Down in GR^{LKO}. **b**, RT-qPCR analysis of representative down- and up-regulated transcripts shown in Fig. 1d, for WT and GR^{LKO} livers, on ctrl. and cal. res. (ZT12, n=4-5). mRNA expression was normalized to *Rplp0*. Box plots display median (central line), 25th-75th percentiles (box), and min-max whiskers, with individual points shown. **c**, Uniform manifold approximation and projection (UMAP) visualization of 11,627 nuclei (n=2/group, 8 samples in total) based on weighted nearest neighbor (WNN) of RNA and ATAC modalities for wildtype (WT) and hepatocyte-specific GR knockouts (GR^{LKO}) mice under control

(ctrl.) and caloric restriction (cal. res.) feeding regimens. **d**, Bar plots showing percentage of cell type in individual samples from WT and GR^{LKO} mice under ctrl. and cal. res. diets. **e**, Bar plots showing the cell cycle analysis in hepatocytes by Cyclone. **f**, Violin plots showing representative expression levels of GR-dependent genes on cal. res. from Fig. 1g. For all violin plots: median (center dot), 25th-75th percentiles (box), min-max whiskers were shown. Source data and exact n per condition are provided in the Source Data file. *$p<0.05$, ** $p<0.01$, and *** $p<0.001$.

Suppl. Fig. 3

a

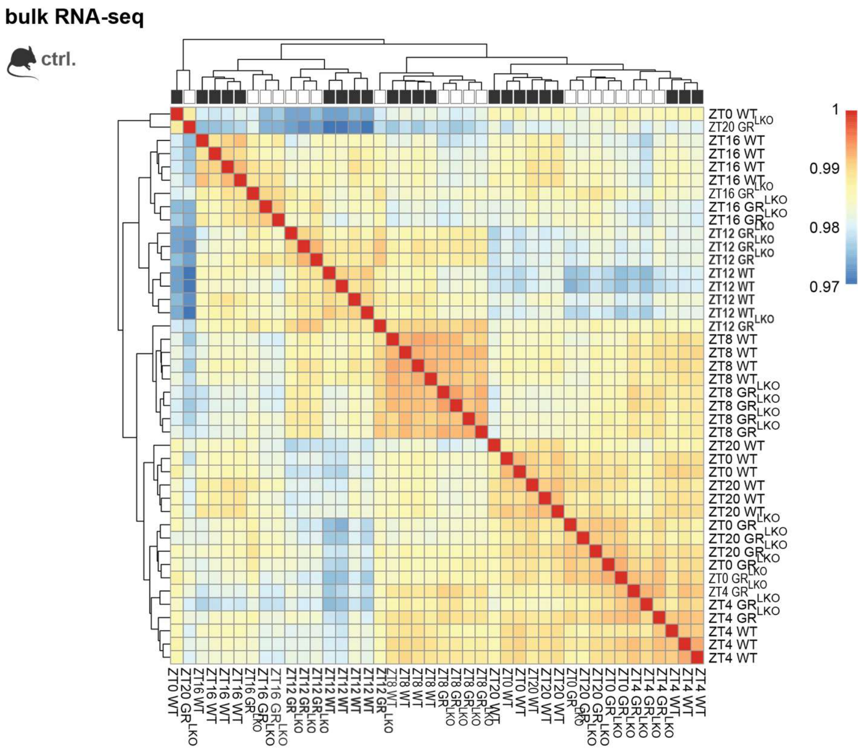

b

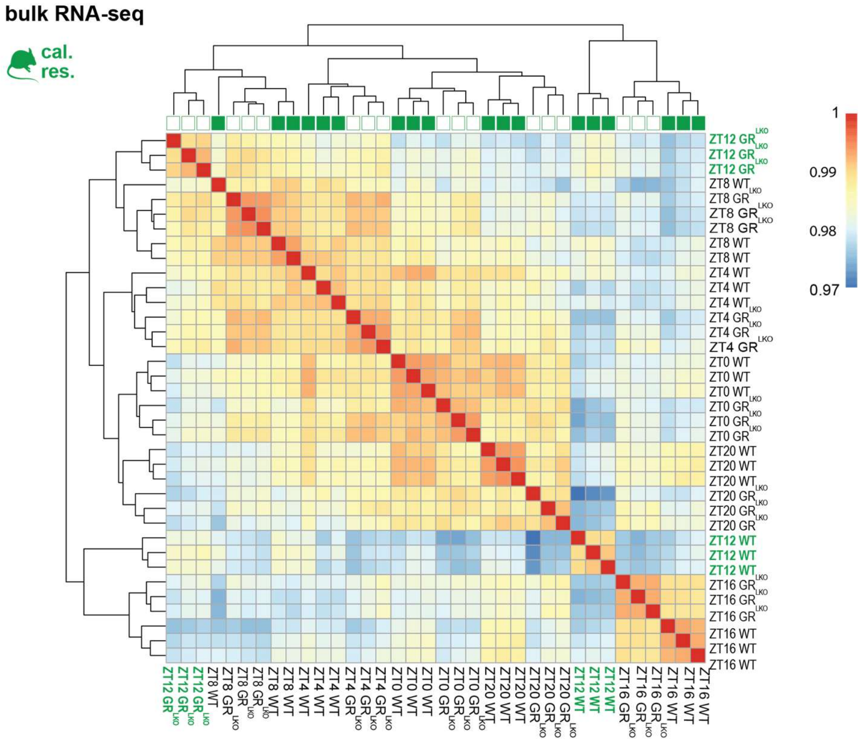

Supplementary Fig. 3, related to Fig. 2

a-b, Unsupervised hierarchical clustering of the normalized RNA-seq VST counts from wildtypes (WT) and hepatocyte-specific GR knockouts (GR^{LKO}) mice after control (ctrl.) (**a**), and caloric restriction (cal. res.) diet (**b**). Samples were collected at six time points: ZT0, ZT4, ZT8, ZT12, ZT16, and ZT20 (n=3-4 per time point).

Suppl. Fig. 4

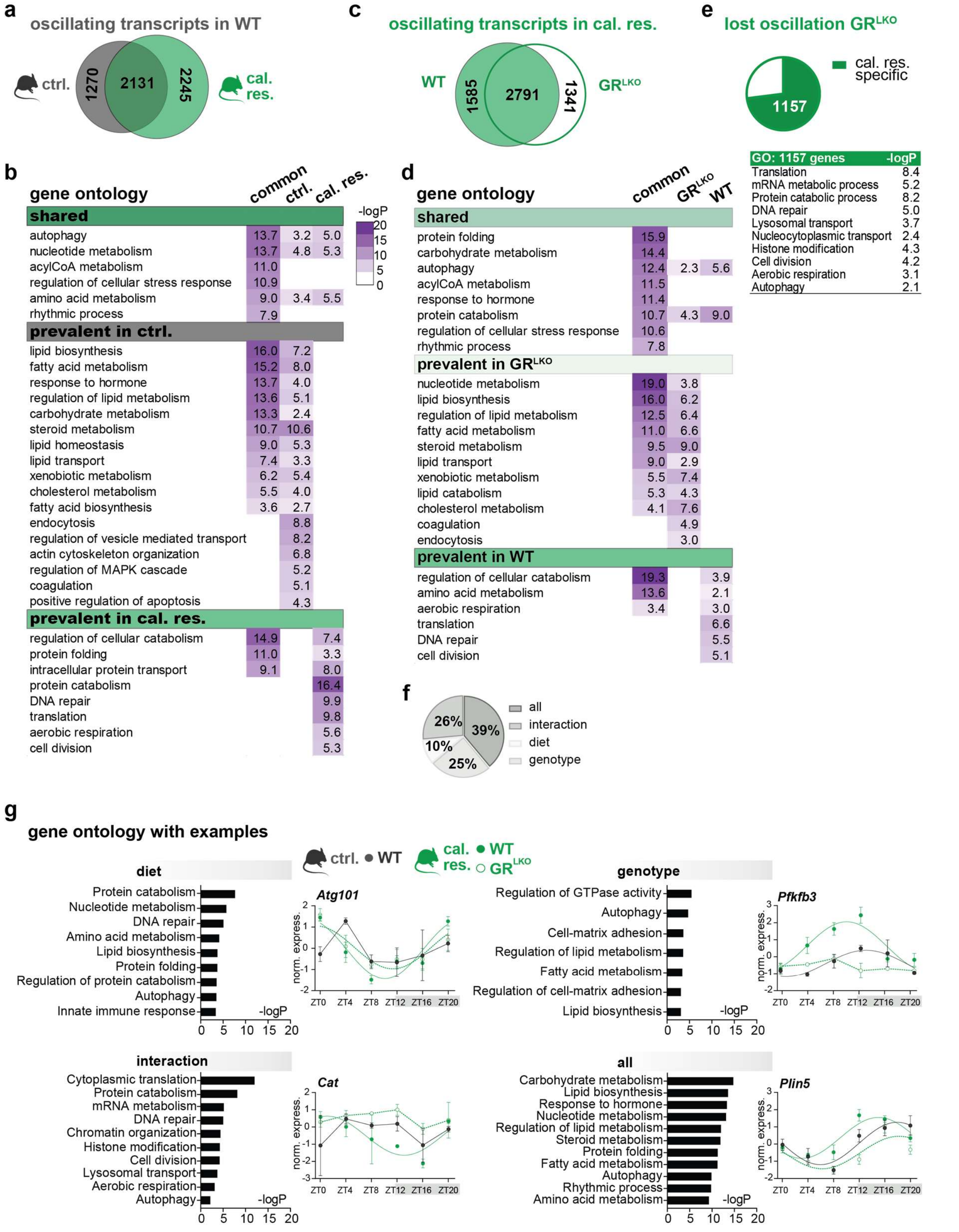

Supplementary Fig. 4, related to Fig. 2

a, Venn diagram showing the number of oscillating transcripts in wildtype (WT) mouse livers subjected to control (ctrl.) and caloric restriction (cal. res.) food regimen (n=3-4). **b**, Gene ontology comparison of common (2,131), cal. res.- specific (2,245), and ctrl.- specific (1,270) oscillating transcripts in WT livers (referring to Fig. 2c). **c**, Venn diagram showing the number of oscillating transcripts in wildtypes

(WT) and hepatocyte-specific GR knockouts (GR^{LKO}) mice after caloric restriction (n=3). **d**, Gene ontology comparison of common (2,791), WT- specific (1,585), and GR^{LKO}- specific (1,341) oscillating transcripts under caloric restriction. **e**, Top: Proportion of de novo oscillating genes under caloric restriction that lose oscillation in GR^{LKO} (GR-dependent cal. res. rhythmicity); Bottom: relative KEGG functional annotation. **f**, Percentages of genes mapping to the four models shown in Fig. 2. **g**, Functional annotation and example genes for the four groups shown in Fig. 2. Expression levels (Z-score normalized read counts) of representative examples for each of the four clusters. Data points represent mean ± SEM (n=3). For transcripts classified as non-rhythmic, data points were connected by simple line plots. For rhythmic ones, a cosinor regression model was applied to visualize oscillatory patterns. Metascape over-representation (hypergeometric) with B-H FDR correction for multiple comparisons was performed for gene ontology. Source data are provided as a Source Data file.

Suppl. Fig. 5

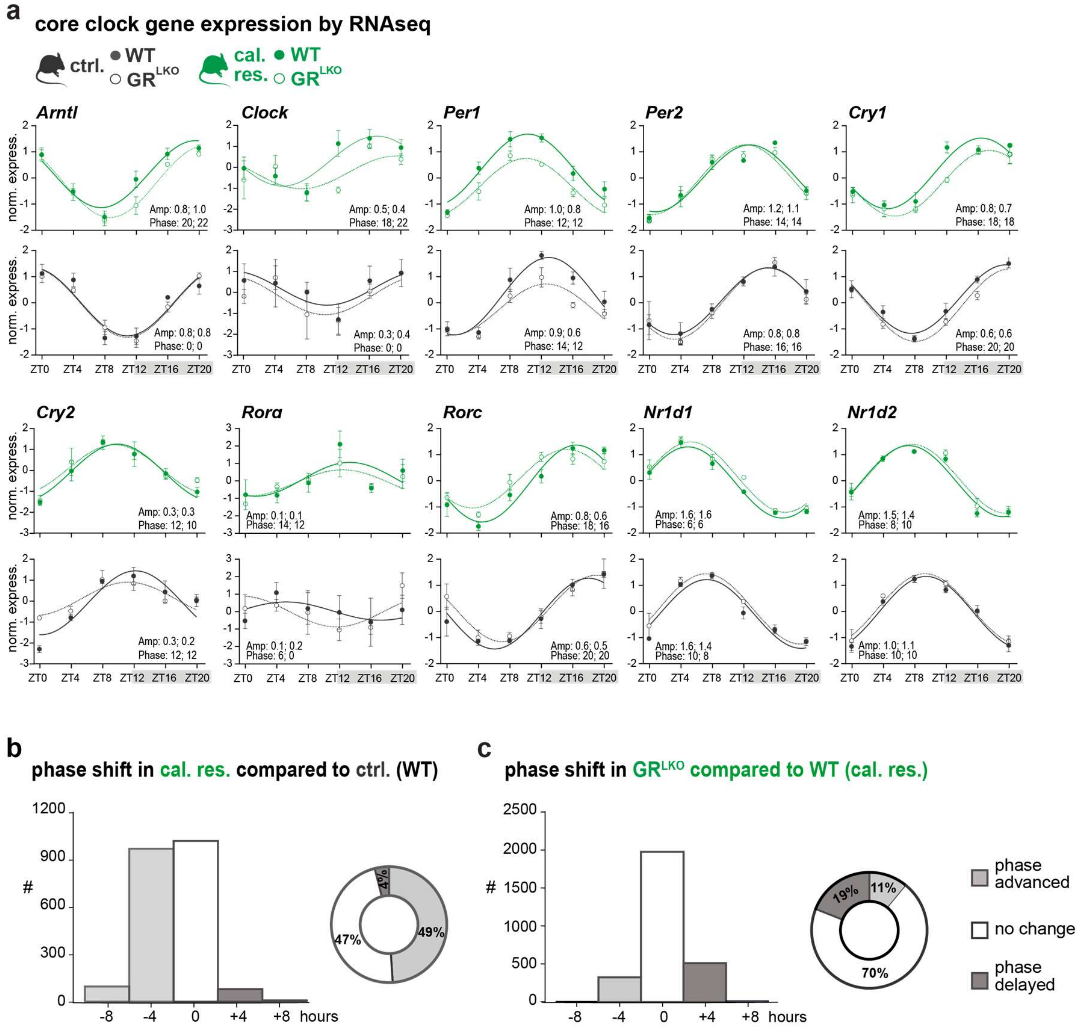

Supplementary Fig. 5, related to Fig. 2

a, Expression levels (z-score normalized read counts) of the core clock genes in livers of wildtypes (WT) and hepatocyte-specific GR knockouts (GR^{LKO}) mice subjected to either caloric restriction (cal. res.) or control (ctrl.) diets. The points show the mean ± SEM (n=3), lines represent cosinor regression fit. Transcript amplitude and phase are noted for each biological group (calculated by JTK_cycle). **b-c**, Histograms and ring charts showing phase shifts, in cal. res. compared to ctrl. -fed mice (**b**), and in GR^{LKO} compared to WT after cal. res. (**c**). Time is binned in 4-hour windows. Source data are provided as a Source Data file.

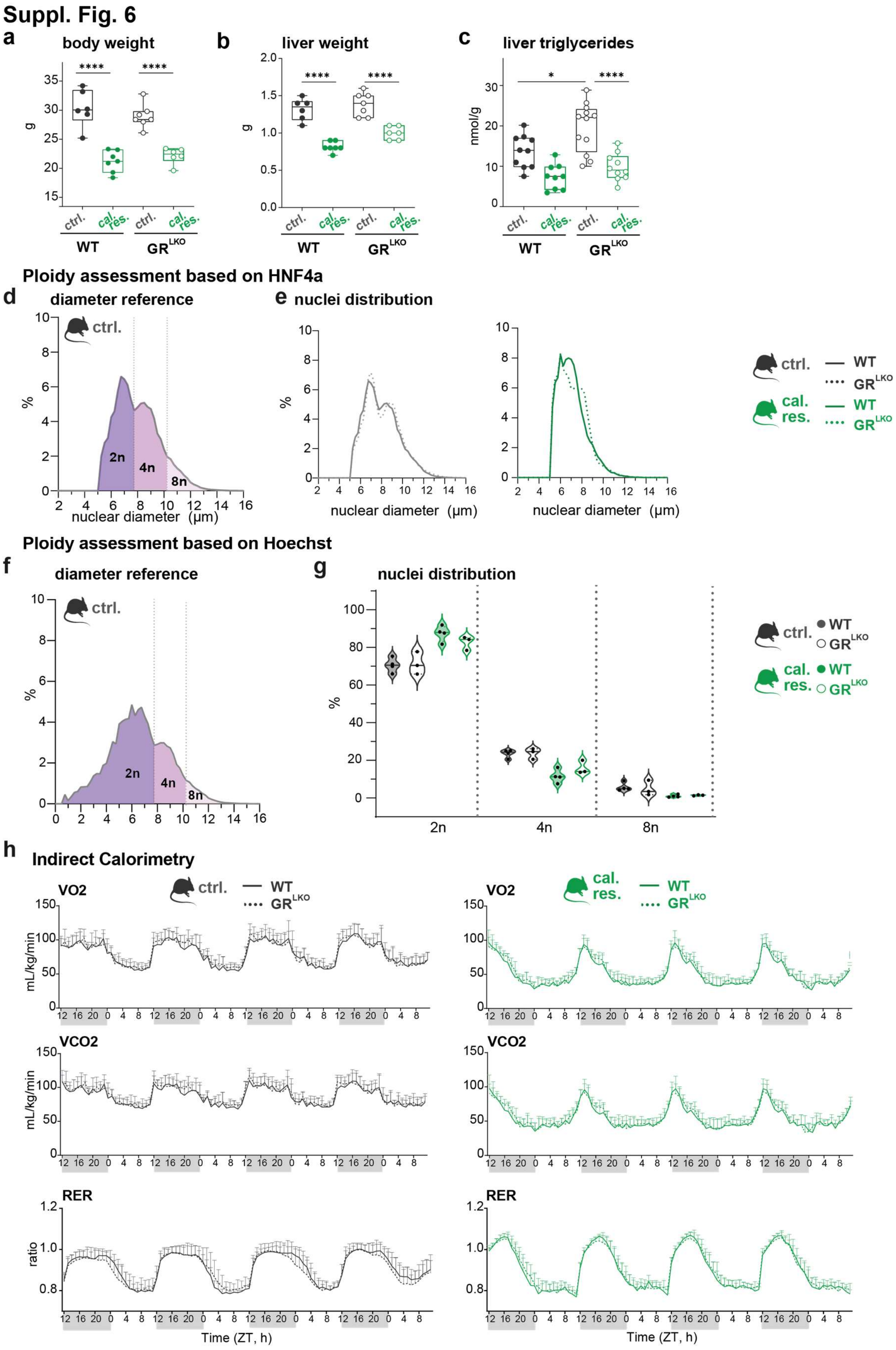

Supplementary Fig. 6, related to Fig. 3

a-c, Whole body weights (n=6-7) (a), liver weights (n=6-7) (b), hepatic triglycerides (n=10-13) (c) in wildtypes (WT) and hepatocyte-specific GR knockouts (GR^{LKO}) mice subjected to control (ctrl.) and

caloric restriction (cal. res.) diet (ZT12). Box plots display median (central line), 25th-75th percentiles (box), and min-max whiskers, with individual points shown. Statistical significance was assessed by two-way ANOVA for multiple comparisons. Asterisks indicate post-hoc multiple comparisons adjusted for multiple testing. **d**, Histogram showing hepatocyte frequency across nuclear diameters based on HNF4α-positive nuclei in ctrl. WT livers, revealing distinct peaks corresponding to diploid (2n), tetraploid (4n), and octaploid (8n) populations. Vertical dashed lines indicate diameter thresholds (7.75 µm and 10.25 µm) used to classify nuclei into ploidy categories. **e**, Histograms showing nuclear diameter distribution of hepatocyte nuclei (HNF4α-positive) comparing WT (solid lines) and GR^{LKO} (dotted lines) mice under ctrl. (gray) and cal. res. (green) conditions**. f,** Histogram showing nuclear diameter distribution of all Hoechst-positive nuclei in ctrl. WT livers. The bimodal distribution reflects the mixed population of predominantly diploid non-parenchymal cells and polyploid hepatocytes. The same diameter thresholds from panel d (7.75 µm and 10.25 µm, vertical dashed lines) were applied for ploidy classification. **g,** Violin plots showing whole-liver ploidy distribution across ploidy categories (2n, 4n, 8n) as percentage of total nuclei, comparing WT (filled symbols) and GR^{LKO} (open symbols) mice under ctrl. (gray) and cal. res. (green) conditions. Horizontal lines indicate median and interquartile range. For d-g, n=3-4 mice per group. **h**, Indirect calorimetry measures (96 h) of VO_2, VCO_2, RER means during the light and dark phase are shown. WT and GR^{LKO} mice were subjected to control (right) and caloric restriction (left) feeding regimen. Each line represents the mean of each respective group (n=7-8/group) measured every 15 min. Source data are provided as a Source Data file. *p<0.05 and ****p<0.0001.

Suppl. Fig. 7

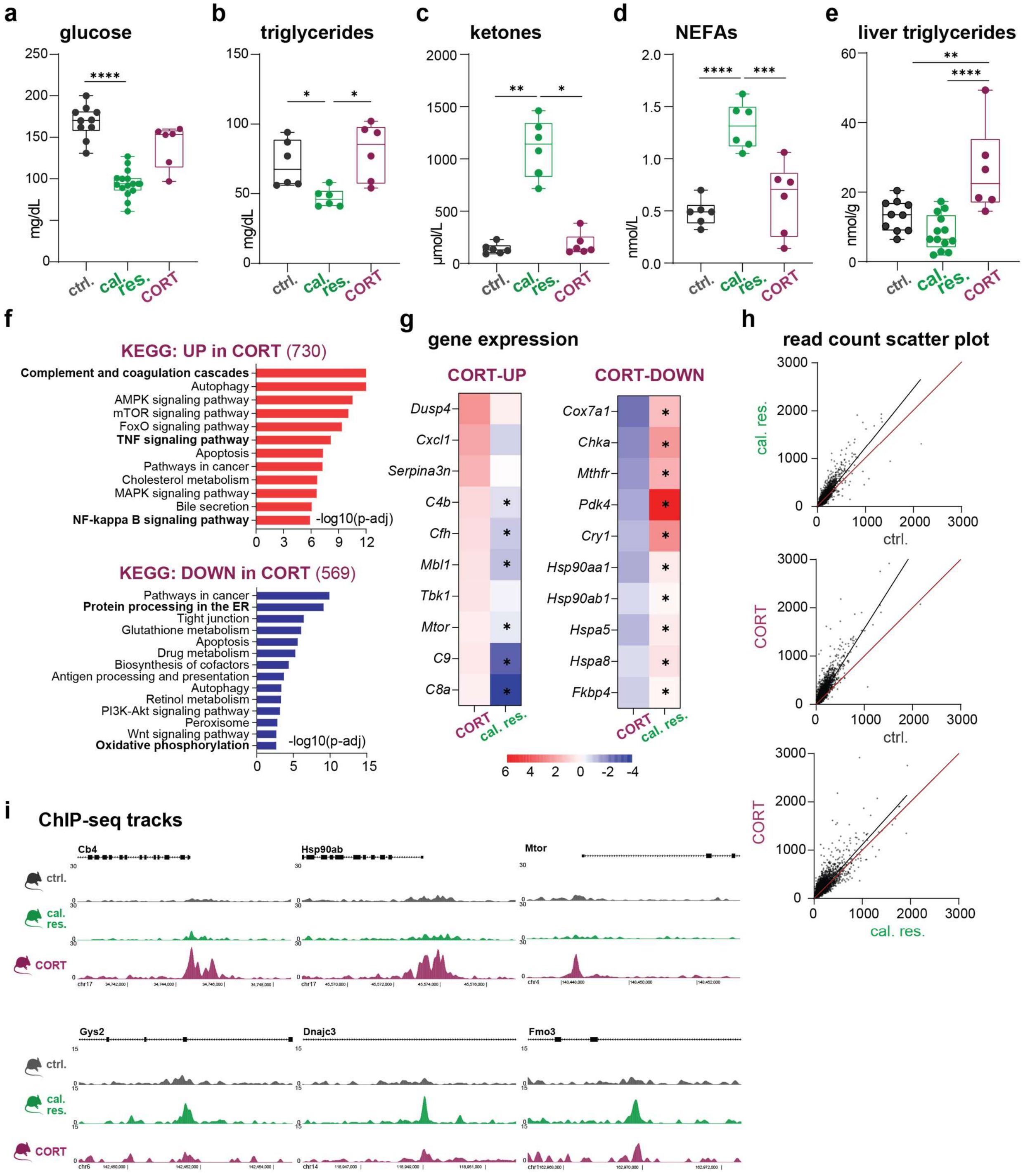

Supplementary Fig. 7, related to Fig. 4

a-e, Serum glucose (n=6-15) (a), triglycerides (n=6) (b), ketone bodies (n=6) (c), non-esterified fatty acids (NEFAs) (n=6) (d), liver triglycerides (n=6-13) (e), measured in wildtype mice subjected to either caloric restriction (cal. res.), control (ctrl.) diet, or control diet plus corticosterone treatment (CORT). Box plots display median (central line), 25th-75th percentiles (box), and min-max whiskers, with individual points shown. Statistical significance was calculated by one-way ANOVA or non-parametric alternatives. Asterisks indicate post-hoc multiple comparisons adjusted for multiple testing. **f**, KEGG

pathway annotation for up- and down-regulated genes in CORT-treated vs. ctrl. mice. g:Profiler (Gene Ontology Statistics) enrichment; p-values adjusted by B-H FDR. **g**, Heatmap showing fold changes in gene expression between 'CORT-treated vs ctrl.' mice and 'cal. res. vs ctrl.' mice (Log2FoldChange) of representative transcripts regulated in opposite directions between CORT-treatment and caloric restriction. Asterisks mark genes significantly deregulated in our caloric restriction data set (Suppl. Fig. 1h). **h,** Read count scatter plots comparing read counts (from ChIP-seq) between experimental conditions. Each dot represents a genomic region belonging to the GR ChIP-seq peak universe. Red diagonal line represents the identity line (x=y); black line indicates the fitted linear regression line. **i,** GR occupancy at representative loci showing increased binding upon CORT treatment (top) or caloric restriction (bottom) as determined by ChIP-seq analysis in mouse livers (normalized tag counts for one representative sample shown). Exact n per conditions are specified in the Source Data file. *$p<0.05$, **$p<0.01$, ***$p<0.001$, and ****$p<0.0001$.

Suppl. Fig. 8

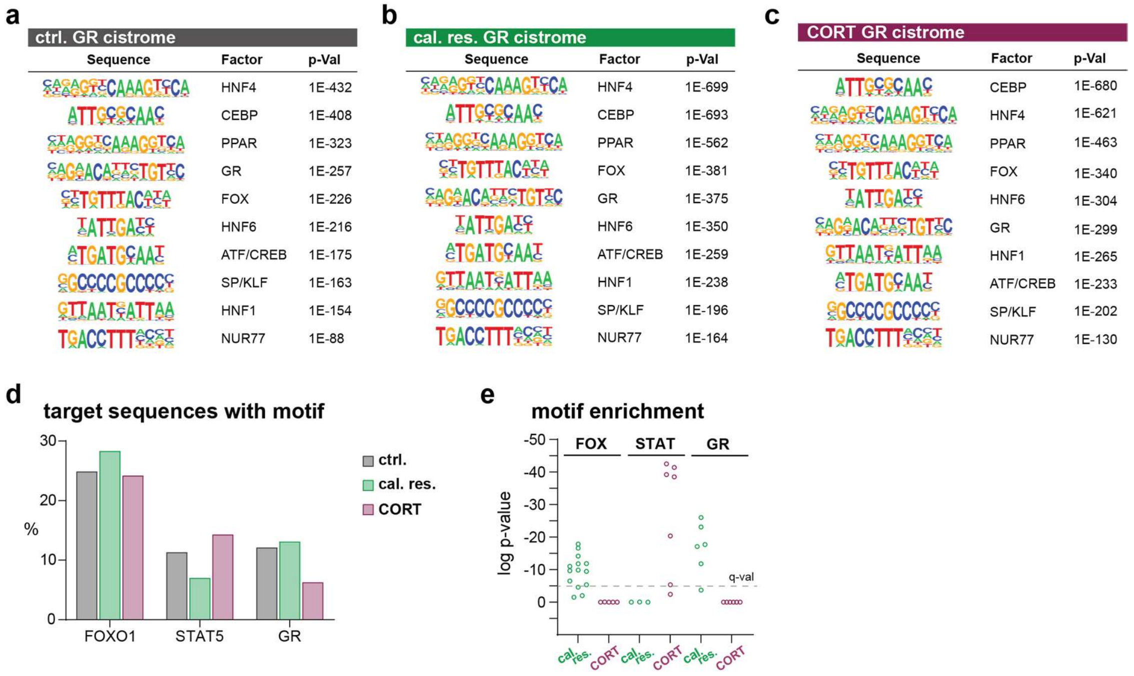

Supplementary Fig. 8, related to Fig. 4

a-c, Top 10 motifs enriched in GR cistromes under control (ctrl.) diet, caloric restriction (cal. res.) diet, and corticosterone (CORT) treatment. HOMER known motif enrichment (binomial test) against default background; raw p-values shown. **d,** Percentage of sequences featuring FoxO1 and STAT5 motifs in control (ctrl.), cal. res-specific, and CORT-specific peaks. **e,** Log-transformed p-value distributions, calculated using reciprocal background analysis for cal. res.- and CORT-specific peaks, are shown. The plotted data represents motif clusters belonging to the Fox, STAT, and GR transcription factor families. Source data are provided as a Source Data file.

Suppl. Fig. 9

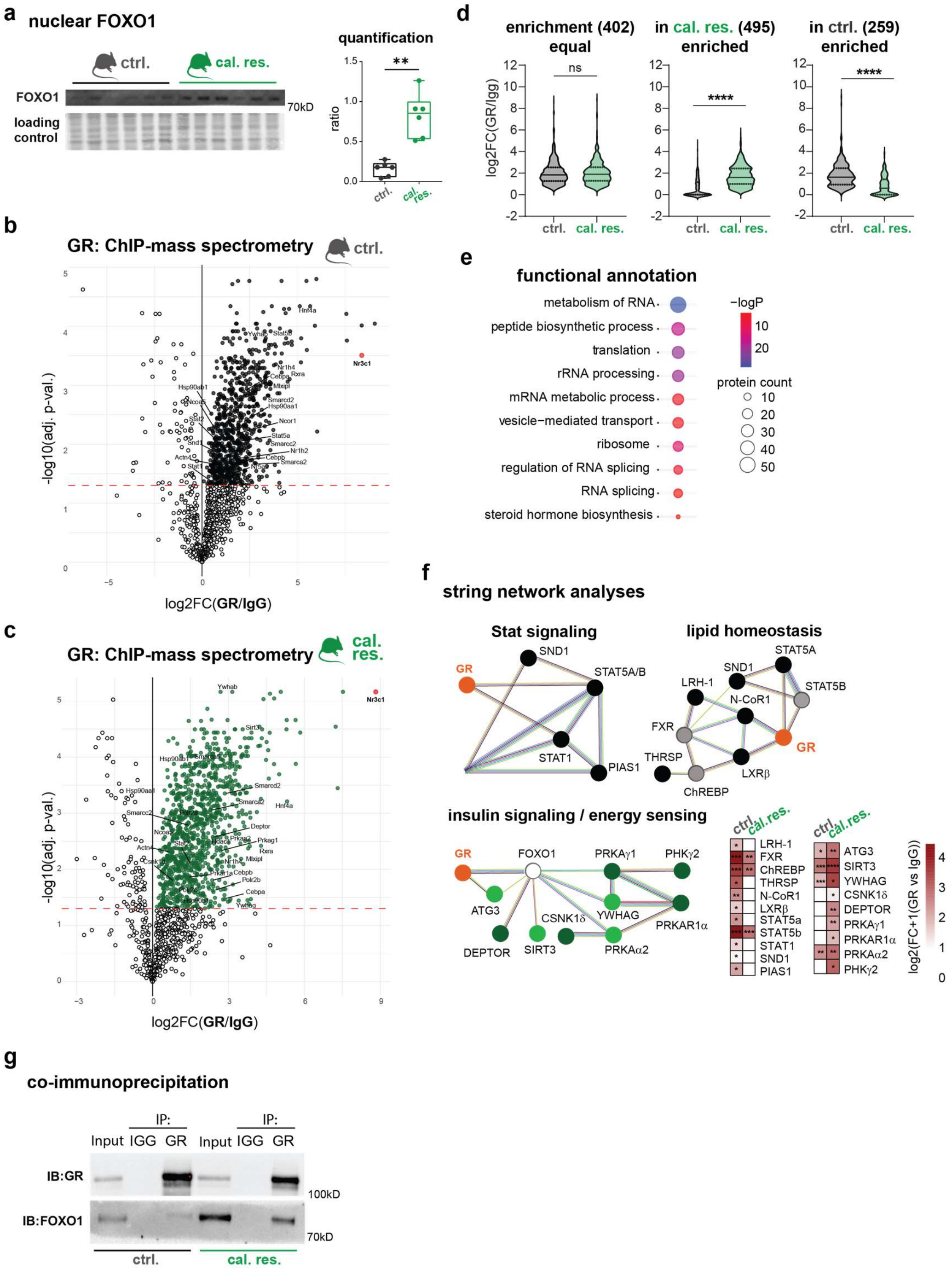

Supplementary Fig. 9, related to Fig. 5

a, Western blot and quantification (immunoblot signal normalized to the loading control) for nuclear FOXO1 in ctrl. and cal. res. wildtype livers collected at ZT12 (n=6). Box plot display median (central line), 25th-75th percentiles (box), and min-max whiskers, with individual points shown. Unpaired t-test was used to compare the two groups. **b,** Volcano plot depicting proteins significantly enriched in the GR interactome under control (ctrl.) diet compared to IgG (n=4, p <0.05, FDR=0.05). Significant proteins are shown with black fill, while non-significant proteins are outlined in black. **c**, Volcano plot depicting proteins significantly enriched in the GR interactome under caloric restriction (cal. res.) diet compared to IgG (n=4, p <0.05, FDR=0.05). Significant proteins are shown with green fill, while non-significant proteins are outlined in black. **d**, Violin plots (median, interquartile range) showing three different

clusters of proteins identified in the interactome based on their enrichment levels. Wilcoxon test was used to compare between the groups. **e**, Functional annotation (Metascape over-representation (hypergeometric) with B-H FDR correction for multiple comparisons) for proteins clustered with equal enrichment, shown in (d). **f**, STRING-generated protein interaction networks for proteins enriched in both ctrl. and cal. res. Heatmap representation of protein enrichment (log2(FC+1(GR vs IgG))) for selected proteins contributing to the protein interaction networks. **g,** Second biological replicate of the immunoprecipitation experiment shown in Fig. 5e. Nuclear extracts were immunoprecipitated using either IgG (negative control) or GR antibody. Western blot analysis was performed with FOXO1 and GR antibodies (IB). Source data are provided as a Source Data file. **p<0.01 and ****p<0.0001.

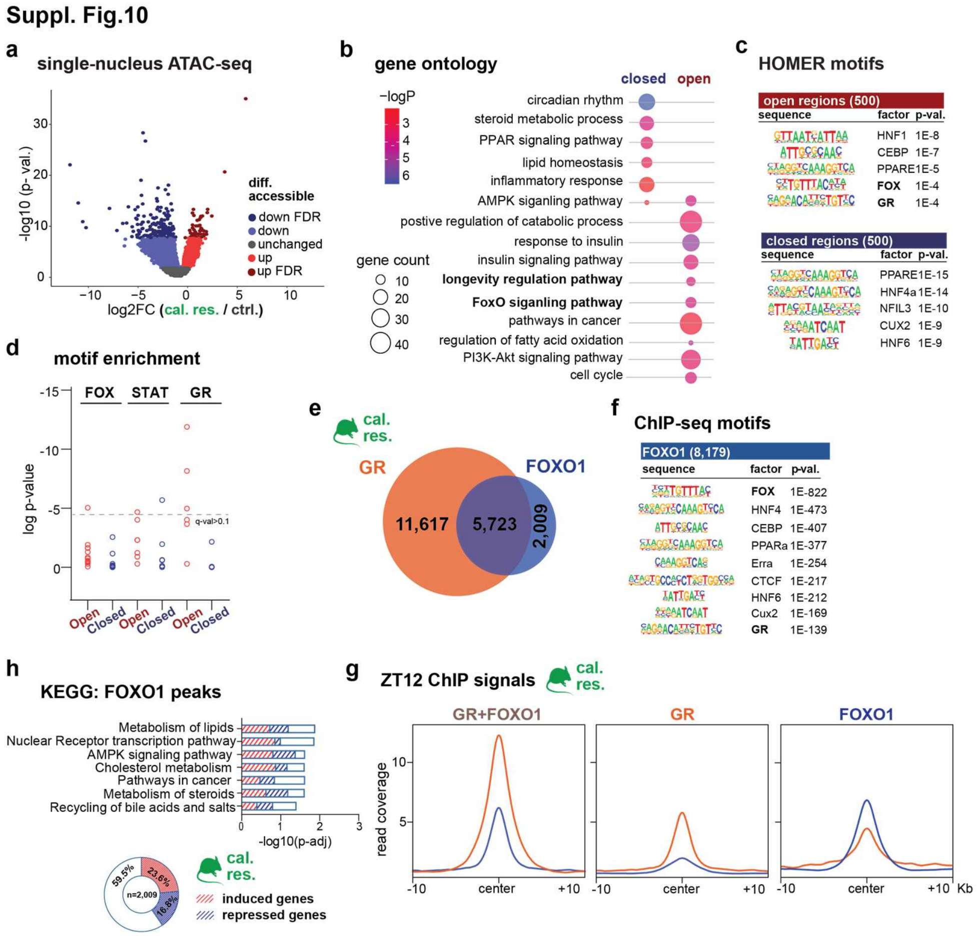

Supplementary Fig. 10, related to Fig. 6

a, Volcano plot of differentially accessible regions (DARs) in cal. res. hepatocytes, compared to control diet (ZT12). Shown in dark blue and red are regions with p-adj < 0.05 for each log2FC directions, while lighter blue and red are regions with p-adj ≥0.05 and p-value <0.05. Differential expression was assessed with Signac using a logistic regression framework. p-values were adjusted for multiple comparisons using Bonferroni correction. **b-c,** Gene ontology (b) and motifs (c) enrichment analysis for the top 500 accessible regions gained (open) or lost (closed) upon caloric restriction. Annotation was performed using Metascape over-representation (hypergeometric) with B-H FDR correction for multiple comparisons. **d,** Log-transformed p-value distributions, calculated using reciprocal background analysis for open and closed regions, are shown. The plotted data represents motif clusters belonging to the Fox, STAT, and GR transcription factor families. **e,** Venn diagram showing the number of overlapping ChIP peaks identified in the GR and FOXO1 cistromes in wildtype mouse livers subjected to caloric restriction (cal. res.). **f**, Top motifs enriched in the FOXO1 cistrome on cal. res., identified by HOMER. **g**, Sequencing tag density of ChIP-seq peaks for overlapping GR and FOXO1 binding sites, and for loci occupied by GR or FOXO1 only (based on the data shown in (e)). **h**, KEGG functional annotation of FOXO1-specific target genes with data integration of RNA-seq profiles (Suppl. Fig. 1h). g:Profiler (Gene Ontology Statistics) enrichment; p-values adjusted by B-H FDR. HOMER known motif enrichment (binomial test) against custom background; raw p-values shown. Source data are provided as a Source Data file.

Supplementary Table 1: List of primers.

Primers sequences used for cloning cis-regulatory regions into the luciferase reporter pGL4.23, for site-directed mutagenesis of predicted FoxO1 binding sites (Fig. 6f) and for mRNA expression analysis by qRT-PCR in mouse livers (Suppl. Fig.2b). In primer identifiers, 'm' represents murine, 'F' and 'R' indicates the forward and reverse primer, respectively.

	**Identifier**	**Sequence (5' to 3')**
**Cloning into pGL4.23**	KpnI-Bcl2l1 F	TTTggtaccGTGTTATCTTTCTTCAGC
	XhoI-Bcl2l1 R	TTTctcgagCCTGGAACTTGTTATATAG
	KpnI-Ppargc1a F	TTTggtaccTCTCTTTGGTTGACTATCC
	XhoI-Ppargc1a R	AAActcgagCATTAAAAAAGGGTCACTG
	NheI-Cry1 F	AAAgctagcCAAATGTGTCCTTGGTACTC
	XhoI-Cry1 R	AAActcgagACTTTGGCTGCTCTCTTAG
**Site-directed mutagenesis**	Bcl2l11-mut F	AAATACTGAGaattCAACATGGGTCCAGC
	Bcl2l11-mut R	GGCAACAGGGTGGATTTTC
	Ppargc1a-mut F	ACTGGCGTAGaattCAAGCACTTCAAATA
	Ppargc1a-mut R	TACATATGTACCTTTTCCAATTG
	Cry1-mut F	AAATCCAGTGaattCTTCCTGC
	Cry1-mut R	CACAAGATCATATTGCTAC
**RT-qPCR**	m*Acaa1* F	GTGGGCAATGTTCTTGAG
	m*Acaa1* R	TGTTGACCGTAGACAAAGG
	m*Bdh2* F	TCAGTTTGCCTCAGAAATCGAT
	m*Bdh2* R	GCCACAGAGGACATGTTGATAA
	m*Bnip3* F	CATGAATCTGGACGAAGTAG
	m*Bnip3* R	AAGCTGTGGCTGTCTATTTC
	m*Cry1* F	ACGTCCCGAGCTGTAGCGGT
	m*Cry1* R	CGCGGAGCTTCTCCCTTGCT
	m*Mat1a* F	GCTCACCTCAAGCAAGACCC
	m*Mat1a* R	CTTGGCAGAGTCGTCATAGC
	m*Pex19* F	CCCGAGCAGTTTGAGAAG
	m*Pex19* R	AGCGAGCCCTCTGAGTAG
	m*Pex5* F	GGAGGTGTCTGGAACTAAAGC
	m*Pex5* R	CAGGGACTCATTGGTAAAGC
	m*Ppargc1a* F	CCCTGCCATTGTTAAGACC
	m*Ppargc1a* R	TGCTGCTGTTCCTGTTTTC
	m*Rplp0* F	AGATTCGGGATATGCTGTTGGC
	m*Rplp0* R	TCGGGTCCTAGACCAGTGTTC
