# Supplementary material for: Hepatic metabolic reprogramming in male mice during short-term caloric restriction involves enhanced glucocorticoid rhythms
Source: Nat Commun. 2025 Dec 11;16:11106. doi: 10.1038/s41467-025-67228-z (PMC12700935; doi:10.1038/s41467-025-67228-z)
Supplement: Supplementary file 2 — Description Of Additional Supplementary File [file 41467_2025_67228_MOESM2_ESM.pdf]

Description of Additional supplementary files

Supplementary Data 1 (XLSX): List of differential transcripts from DESeq2 and JTK Cycle Analyses.

Supplementary Data 2 (XLSX): Summary statistics of identified cell types from singlenucleus multiome dataset. In hepatocytes, includes the results of MAST-based differential gene expression and the Differentially Accessible Regions (DARs) analyses.

Supplementary Data 3 (XLSX): GR, and FOXO1 annotated ChIP-Seq peaks in ctrl. and cal. res. livers.

Supplementary Data 4 (XLSX): GR-enriched proteins from ChIP-MS in ctrl. and cal. res. conditions. Includes additional protein clustering based on enrichment patterns and comparative enrichment visualization.
