# Supplementary material for: Hepatic metabolic reprogramming in male mice during short-term caloric restriction involves enhanced glucocorticoid rhythms
Source: Nat Commun. 2025 Dec 11;16:11106. doi: 10.1038/s41467-025-67228-z (PMC12700935; doi:10.1038/s41467-025-67228-z)
Supplement: Supplementary file 7 — Reporting summary [file 41467_2025_67228_MOESM7_ESM.pdf]

nature portfolio

Corresponding author(s): Fabiana Quagliarini

Last updated by author(s): Nov 20, 2025

Reporting Summary

Nature Portfolio wishes to improve the reproducibility of the work that we publish. This form provides structure for consistency and transparency in reporting. For further information on Nature Portfolio policies, see our Editorial Policies and the Editorial Policy Checklist.

Statistics

For all statistical analyses, confirm that the following items are present in the figure legend, table legend, main text, or Methods section.

n/a	Confirmed	
☐	☒	The exact sample size (n) for each experimental group/condition, given as a discrete number and unit of measurement
☐	☒	A statement on whether measurements were taken from distinct samples or whether the same sample was measured repeatedly
☐	☒	The statistical test(s) used AND whether they are one- or two-sided<br>*Only common tests should be described solely by name; describe more complex techniques in the Methods section.*
☒	☐	A description of all covariates tested
☐	☒	A description of any assumptions or corrections, such as tests of normality and adjustment for multiple comparisons
☐	☒	A full description of the statistical parameters including central tendency (e.g. means) or other basic estimates (e.g. regression coefficient) AND variation (e.g. standard deviation) or associated estimates of uncertainty (e.g. confidence intervals)
☐	☒	For null hypothesis testing, the test statistic (e.g. F, t, r) with confidence intervals, effect sizes, degrees of freedom and P value noted<br>*Give P values as exact values whenever suitable.*
☒	☐	For Bayesian analysis, information on the choice of priors and Markov chain Monte Carlo settings
☒	☐	For hierarchical and complex designs, identification of the appropriate level for tests and full reporting of outcomes
☐	☒	Estimates of effect sizes (e.g. Cohen's d, Pearson's r), indicating how they were calculated

Our web collection on statistics for biologists contains articles on many of the points above.

Software and code

Policy information about availability of computer code

Data collection	Data collection is described in Methods section. No custom software was used.
Data analysis	All data analysis is detailed in the Methods section.<br><br>Western blot images were quantified using ImageJ 1.53e and assembled in Adobe Illustrator version 28.0.<br><br>Serum measurements, ELISA data, and colorimetric assay results were calculated using Excel for Microsoft 365 MSO version 2408.<br><br>Bioanalyzer data was processed using 2100 Expert Software Revision B.02.12.<br><br>Ploidy measurements from scanned liver sections were analyzed using Visiopharm software (Version 2023.09.01 4530).<br><br>Statistical analyses and graphical visualizations were performed using R version 4.3.1, RStudio version 4.0.1, or GraphPad Prism version 10.<br><br>Confocal images of Autophagy reporter were analysed using Olympus Fluoview version 4.2b software.<br><br>Confocal images of FOXO1 immunofluorescence were analysed using ImageJ 1.53c software.<br><br>RNAseq analysis: FastQC ver. 0.11.9, Salmon ver. 1.8.0, MultiQC ver. 1.12, R/RStudio ver. 4.0.1 with DESeq2 package ver. 1.30.5 and Metacycle package for differential rhythmicity analysis, GraphPad Prism version 10.

Single-nuclei multiome (RNA-seq and ATAC-seq) analysis:CellRanger ARC 2.0.2, R version 4.3.2 with Seurat version 5.1.0, Signac version 1.13.0, and Scran package, MACS2 version 2.2.6.

ChIP-MS analysis: MaxQuant v1.6.15.0, Python with custom analysis pipeline based on Clinical Knowledge Graph, R with ggplot2 and pheatmap packages, GraphPad Prism version 10, Metascape and gProfiler web-based tools, STRING database.

Custom code: No custom code was generated for this study. All analyses used standard software packages as detailed above.

For manuscripts utilizing custom algorithms or software that are central to the research but not yet described in published literature, software must be made available to editors and reviewers. We strongly encourage code deposition in a community repository (e.g. GitHub). See the Nature Portfolio guidelines for submitting code & software for further information.

Data

Policy information about availability of data

All manuscripts must include a data availability statement. This statement should provide the following information, where applicable:

- Accession codes, unique identifiers, or web links for publicly available datasets
- A description of any restrictions on data availability
- For clinical datasets or third party data, please ensure that the statement adheres to our policy

All sequencing data generated in this study have been deposited in the Gene Expression Omnibus (GEO) database under accession code GSE248866 [GEO Accession viewer]. The mass spectrometry proteomics data have been deposited to the ProteomeXchange Consortium via the PRIDE partner repository with the dataset identifier PXD070287 [https://www.ebi.ac.uk/pride/archive/projects/PXD070287]. The processed results are included as Supplementary Data. Other data generated in this study are provided in the Supplementary Information/Source Data file. All data supporting the findings described in this manuscript are also available from the corresponding authors upon request.

Research involving human participants, their data, or biological material

Policy information about studies with human participants or human data. See also policy information about sex, gender (identity/presentation), and sexual orientation and race, ethnicity and racism.

Reporting on sex and gender	*Use the terms sex (biological attribute) and gender (shaped by social and cultural circumstances) carefully in order to avoid confusing both terms. Indicate if findings apply to only one sex or gender; describe whether sex and gender were considered in study design; whether sex and/or gender was determined based on self-reporting or assigned and methods used.*<br>*Provide in the source data disaggregated sex and gender data, where this information has been collected, and if consent has been obtained for sharing of individual-level data; provide overall numbers in this Reporting Summary. Please state if this information has not been collected.*<br>*Report sex- and gender-based analyses where performed, justify reasons for lack of sex- and gender-based analysis.*
Reporting on race, ethnicity, or other socially relevant groupings	*Please specify the socially constructed or socially relevant categorization variable(s) used in your manuscript and explain why they were used. Please note that such variables should not be used as proxies for other socially constructed/relevant variables (for example, race or ethnicity should not be used as a proxy for socioeconomic status).*<br>*Provide clear definitions of the relevant terms used, how they were provided (by the participants/respondents, the researchers, or third parties), and the method(s) used to classify people into the different categories (e.g. self-report, census or administrative data, social media data, etc.)*<br>*Please provide details about how you controlled for confounding variables in your analyses.*
Population characteristics	*Describe the covariate-relevant population characteristics of the human research participants (e.g. age, genotypic information, past and current diagnosis and treatment categories). If you filled out the behavioural & social sciences study design questions and have nothing to add here, write "See above."*
Recruitment	*Describe how participants were recruited. Outline any potential self-selection bias or other biases that may be present and how these are likely to impact results.*
Ethics oversight	*Identify the organization(s) that approved the study protocol.*

Note that full information on the approval of the study protocol must also be provided in the manuscript.

Field-specific reporting

Please select the one below that is the best fit for your research. If you are not sure, read the appropriate sections before making your selection.

☒ Life sciences ☐ Behavioural & social sciences ☐ Ecological, evolutionary & environmental sciences

For a reference copy of the document with all sections, see nature.com/documents/nr-reporting-summary-flat.pdf

Life sciences study design

All studies must disclose on these points even when the disclosure is negative.

Sample size	No calculations were done to determine sample size.

Sample size	For phenotypical characterization sample size was determined based on standard animal studies, assuring a minimum of n = 3 biological replicates with sufficient reproducibility.<br><br>Imaging: Immunofluorescence of FOXO1 in primary hepatocytes was performed across two independent experiments, with a total of 86-126 cells analyzed. For autophagy reporter, experiments were performed three times with 19-50 cells analyzed per condition. Ploidy was assessed from WT ctrl. n=4, WT cal.res. n=4, GRLKO ctrl. n=3, GRLKO cal.res. n=3.<br><br>Single-nuclei multiome was performed using 2 biological replicates.<br><br>ChIP-MS and RNA-seq were performed using 3 to 4 biological replicates.<br><br>ChIP-seq and co-immunoprecipitations included 2 biological replicates.<br><br>Luciferase assays included 3 independent biological replicates for WT reporters (n=3) and 2 independent biological replicates for mutant reporters (n=2), with WT included as an internal control in experiments containing mutants.
Data exclusions	Phenotypical data points were excluded by performing outlier test in case of finding an unusual value as described in the Methods.
Replication	All attempts at replication were successful.
Randomization	There was a random allocation between experimental groups.
Blinding	Blinding was not possible in order to collect all the time points between the two distinict genotypes, having enough biological and technical rounds.

Behavioural & social sciences study design

All studies must disclose on these points even when the disclosure is negative.

Study description	*Briefly describe the study type including whether data are quantitative, qualitative, or mixed-methods (e.g. qualitative cross-sectional, quantitative experimental, mixed-methods case study).*
Research sample	*State the research sample (e.g. Harvard university undergraduates, villagers in rural India) and provide relevant demographic information (e.g. age, sex) and indicate whether the sample is representative. Provide a rationale for the study sample chosen. For studies involving existing datasets, please describe the dataset and source.*
Sampling strategy	*Describe the sampling procedure (e.g. random, snowball, stratified, convenience). Describe the statistical methods that were used to predetermine sample size OR if no sample-size calculation was performed, describe how sample sizes were chosen and provide a rationale for why these sample sizes are sufficient. For qualitative data, please indicate whether data saturation was considered, and what criteria were used to decide that no further sampling was needed.*
Data collection	*Provide details about the data collection procedure, including the instruments or devices used to record the data (e.g. pen and paper, computer, eye tracker, video or audio equipment) whether anyone was present besides the participant(s) and the researcher, and whether the researcher was blind to experimental condition and/or the study hypothesis during data collection.*
Timing	*Indicate the start and stop dates of data collection. If there is a gap between collection periods, state the dates for each sample cohort.*
Data exclusions	*If no data were excluded from the analyses, state so OR if data were excluded, provide the exact number of exclusions and the rationale behind them, indicating whether exclusion criteria were pre-established.*
Non-participation	*State how many participants dropped out/declined participation and the reason(s) given OR provide response rate OR state that no participants dropped out/declined participation.*
Randomization	*If participants were not allocated into experimental groups, state so OR describe how participants were allocated to groups, and if allocation was not random, describe how covariates were controlled.*

Ecological, evolutionary & environmental sciences study design

All studies must disclose on these points even when the disclosure is negative.

Study description	*Briefly describe the study. For quantitative data include treatment factors and interactions, design structure (e.g. factorial, nested, hierarchical), nature and number of experimental units and replicates.*
Research sample	*Describe the research sample (e.g. a group of tagged Passer domesticus, all Stenocereus thurberi within Organ Pipe Cactus National Monument), and provide a rationale for the sample choice. When relevant, describe the organism taxa, source, sex, age range and any manipulations. State what population the sample is meant to represent when applicable. For studies involving existing datasets, describe the data and its source.*

Sampling strategy	*Note the sampling procedure. Describe the statistical methods that were used to predetermine sample size OR if no sample-size calculation was performed, describe how sample sizes were chosen and provide a rationale for why these sample sizes are sufficient.*
Data collection	*Describe the data collection procedure, including who recorded the data and how.*
Timing and spatial scale	*Indicate the start and stop dates of data collection, noting the frequency and periodicity of sampling and providing a rationale for these choices. If there is a gap between collection periods, state the dates for each sample cohort. Specify the spatial scale from which the data are taken*
Data exclusions	*If no data were excluded from the analyses, state so OR if data were excluded, describe the exclusions and the rationale behind them, indicating whether exclusion criteria were pre-established.*
Reproducibility	*Describe the measures taken to verify the reproducibility of experimental findings. For each experiment, note whether any attempts to repeat the experiment failed OR state that all attempts to repeat the experiment were successful.*
Randomization	*Describe how samples/organisms/participants were allocated into groups. If allocation was not random, describe how covariates were controlled. If this is not relevant to your study, explain why.*
Blinding	*Describe the extent of blinding used during data acquisition and analysis. If blinding was not possible, describe why OR explain why blinding was not relevant to your study.*

Did the study involve field work? ☐ Yes ☐ No

Field work, collection and transport

Field conditions	*Describe the study conditions for field work, providing relevant parameters (e.g. temperature, rainfall).*
Location	*State the location of the sampling or experiment, providing relevant parameters (e.g. latitude and longitude, elevation, water depth).*
Access & import/export	*Describe the efforts you have made to access habitats and to collect and import/export your samples in a responsible manner and in compliance with local, national and international laws, noting any permits that were obtained (give the name of the issuing authority, the date of issue, and any identifying information).*
Disturbance	*Describe any disturbance caused by the study and how it was minimized.*

Reporting for specific materials, systems and methods

We require information from authors about some types of materials, experimental systems and methods used in many studies. Here, indicate whether each material, system or method listed is relevant to your study. If you are not sure if a list item applies to your research, read the appropriate section before selecting a response.

Materials & experimental systems

n/a	Involved in the study	
☐	☒	Antibodies
☐	☒	Eukaryotic cell lines
☒	☐	Palaeontology and archaeology
☐	☒	Animals and other organisms
☒	☐	Clinical data
☒	☐	Dual use research of concern
☒	☐	Plants

Methods

n/a	Involved in the study	
☐	☒	ChIP-seq
☒	☐	Flow cytometry
☒	☐	MRI-based neuroimaging

Antibodies

Antibodies used

Western Blot Primary Antibodies:
Rabbit anti-GR (Cell Signaling, #12041) 1:1000
Rabbit anti-LC3A/B (Cell Signaling, #12741) 1:1000
Rabbit anti-FOXO1 (Ser256) (Cell Signaling, #9461) 1:1000
Rabbit anti-STAT5 A,B (pY694, pY699) (Cell Signaling, #9351) 1:1000
Mouse anti-GR (Santa Cruz, #sc-393232) 1:5000
Mouse anti-FOXO1 (Cell Signaling, #97635) 1:1000

Western Blot Secondary Antibodies:
Goat anti-Mouse IgG HRP-conjugated (BioRad, #1706516) 1:5000
Goat anti-Rabbit IgG HRP-conjugated (Dako, #P044801-2) 1:5000

Rabbit anti-IgG HRP-linked (Cell Signaling, #7074) 1:5000

Co-Immunoprecipitation:
Rabbit anti-GR (ProteinTech, #24050-1-AP) 3 μg
Rabbit anti-IgG (Cell Signaling, #2729) 3 μg

Immunofluorescence in Primary Hepatocytes:
Rabbit anti-FOXO1 (ProteinTech, #18592-1-AP) 1:150
Alexa Fluor 568 goat anti-rabbit secondary (Invitrogen, #A11011) 2 μg/ml
Alexa Fluor 488 phalloidin (Invitrogen, #A12379) 1:400

Immunofluorescence for Ploidy Measurement:
Goat polyclonal anti-β-catenin (R&D Systems, #AF1392) 1:20
Rabbit anti-HNF4α mAb (Cell Signaling, #3113) 1:50
AlexaFluor 550-conjugated donkey anti-goat IgG (Invitrogen, #A21432) 1:100
AlexaFluor 647-conjugated donkey anti-rabbit IgG (Invitrogen, #A31573) 1:100

ChIP-seq for GR:
Rabbit anti-GR (ProteinTech, #24050-1-AP) 8 μg

ChIP-seq for FOXO1:
Rabbit anti-FOXO1 (ProteinTech, #18592-1-AP) 4 μg
Rabbit anti-FOXO1 (Abcam, #ab39670) 4 μg

ChIP-MS:
Rabbit anti-GR (ProteinTech, #24050-1-AP) 6 μg
Rabbit anti-IgG (Cell Signaling, #2729) 6 μg

Validation

All antibodies have been validated by the manufacturers and/or in peer-reviewed publications:

Rabbit anti-GR (Cell Signaling, #12041): Recognizes endogenous levels of total GR protein. This antibody reacts with GR-α and GR-β but does not cross-react with mineralocorticoid receptor. Validated for Western Blot: https://www.cellsignal.com/products/primary-antibodies/glucocorticoid-receptor-d6h2l-xp-rabbit-mab/12041

Rabbit anti-LC3A/B (Cell Signaling, #12741): Recognizes endogenous levels of total LC3A and LC3B proteins. Validated for Western Blot and IF: https://www.cellsignal.com/products/primary-antibodies/lc3a-b-d3u4c-xp-rabbit-mab/12741

Rabbit anti-Phospho-FOXO1 (Ser256) (Cell Signaling, #9461): Detects endogenous levels of FOXO1 only when phosphorylated at serine 256. The antibody cross-reacts with FOXO4 phosphorylated at Ser193. Validated for Western Blot: https://www.cellsignal.com/products/primary-antibodies/phospho-foxo1-ser256-antibody/9461. Additional validation: Zhihua Sun et al., https://doi.org/10.1155/2020/1484321

Rabbit anti-phospho-STAT5 A,B (pY694, pY699) (Cell Signaling, #9351): Detects endogenous levels of STAT5a only when phosphorylated at Tyr694 and STAT5b when phosphorylated at Tyr699. Does not cross-react with the corresponding phospho-tyrosine residues of other STAT proteins, but does cross-react with EGFR. Validated for Western Blot: https://www.cellsignal.com/products/primary-antibodies/phospho-stat5-tyr694-antibody/9351

Mouse anti-GR (Santa Cruz, #sc-393232): Recognizes GR-α and GR-β. Validated for Western Blot: https://www.scbt.com/p/gr-antibody-g-5

Mouse anti-FOXO1 (Cell Signaling, #97635): Recognizes endogenous levels of total FOXO1 protein. Does not detect exogenously expressed family members FOXO3a or FOXO4. Validated for Western Blot: https://www.cellsignal.com/products/primary-antibodies/foxo1-d8t1s-mouse-mab/97635

Rabbit anti-GR (ProteinTech, #24050-1-AP): Targets glucocorticoid receptor in WB, IHC, IF, IP, CoIP, ChIP, and ELISA applications with reactivity in human, mouse, and rat samples. Validated for IP and ChIP: https://www.ptglab.com/Products/NR3C1-Antibody-24050-1-AP.htm#publications. Additional validation: Quagliarini et al., DOI: 10.1016/j.molcel.2019.10.007

Rabbit anti-IgG (Cell Signaling, #2729): Unconjugated rabbit polyclonal antibody routinely used as a non-specific IgG control in chromatin immunoprecipitation and immunoprecipitation. Validated for ChIP and IP: https://www.cellsignal.com/products/primary-antibodies/normal-rabbit-igg/2729

Rabbit anti-FOXO1 (ProteinTech, #18592-1-AP): Used for IF in primary murine hepatocytes and ChIP-seq in frozen murine livers. Validated applications: https://www.thermofisher.com/antibody/product/FOXO1-Antibody-Polyclonal/18592-1-AP

Rabbit anti-FOXO1 (Abcam, #ab39670): Used in cocktail with ProteinTech #18592-1-AP for ChIP-seq in frozen murine livers. Validated applications: https://www.abcam.com/en-us/products/primary-antibodies/foxo1a-antibody-ab39670

Goat polyclonal anti-β-catenin (R&D Systems, #AF1392): Used for ploidy measurement immunofluorescence. Validated by manufacturer.

Rabbit anti-HNF4α mAb (Cell Signaling, #3113): Used for ploidy measurement immunofluorescence. Validated by manufacturer.

Eukaryotic cell lines

Policy information about cell lines and Sex and Gender in Research

Cell line source(s)	Hepatocellular Carcinoma cells (HepG2)-ATCC HB-8065<br>CV1 cells: Salk Institute.
Authentication	None of the cell lines are authenticated.
Mycoplasma contamination	HepG2 were tested negative, while CV1 cells were tested positive.
Commonly misidentified lines<br>(See ICLAC register)	No misidentified lines were used.

Palaeontology and Archaeology

Specimen provenance	*Provide provenance information for specimens and describe permits that were obtained for the work (including the name of the issuing authority, the date of issue, and any identifying information). Permits should encompass collection and, where applicable, export.*
Specimen deposition	*Indicate where the specimens have been deposited to permit free access by other researchers.*
Dating methods	*If new dates are provided, describe how they were obtained (e.g. collection, storage, sample pretreatment and measurement), where they were obtained (i.e. lab name), the calibration program and the protocol for quality assurance OR state that no new dates are provided.*

☐ Tick this box to confirm that the raw and calibrated dates are available in the paper or in Supplementary Information.

Ethics oversight	*Identify the organization(s) that approved or provided guidance on the study protocol, OR state that no ethical approval or guidance was required and explain why not.*

Note that full information on the approval of the study protocol must also be provided in the manuscript.

Animals and other research organisms

Policy information about studies involving animals; ARRIVE guidelines recommended for reporting animal research, and Sex and Gender in Research

Laboratory animals	Male C57BL/6J mice 20-21 weeks of age were used for this study. WT=76; GRfl/fl + GRfl/fl x AlbCRE = 113. Animals were housed in a controlled environment (12h light/12h dark daily cycle, 20-24 °C, and 45-65% humidity) and fed with chow diet (Altromin GmbH, #1314).<br>Literature for GRfl/fl + GRfl/fl x AlbCRE mice<br>Quagliarini, F. et al. Cistromic Reprogramming of the Diurnal Glucocorticoid Hormone Response by High-Fat Diet. Mol Cell 76, 531-545.e535 (2019).<br>Opherk, C. et al. Inactivation of the glucocorticoid receptor in hepatocytes leads to fasting hypoglycemia and ameliorates hyperglycemia in streptozotocin-induced diabetes mellitus. Mol Endocrinol 18, 1346-1353 (2004).
Wild animals	Study did not involve wild animals.
Reporting on sex	The findings apply to only male C57BL/6J mice. Sex was considered in the study design - only male mice were used to minimize confounding variables in metabolic experiments. Sex was not deliberately omitted but focused on one sex to ensure consistency across measurements and reduce variability in body measurments and metabolic analyses.
Field-collected samples	The study did not involve samples collected from the field.
Ethics oversight	All mouse experiments were performed according to the rules and guidelines established by the Institutional Animal Committee at Helmholtz Munich Center. Ethical approval was received from the local animal welfare authority (District government of upper Bavaria; ROB- 55.2-2532.Vet_02-19-43, ROB- 55.2-2532.Vet_02-19-80, and ROB- 55.2-2532.Vet_02-24-16).

Note that full information on the approval of the study protocol must also be provided in the manuscript.

Clinical data

Policy information about clinical studies

All manuscripts should comply with the ICMJE guidelines for publication of clinical research and a completed CONSORT checklist must be included with all submissions.

Clinical trial registration	*Provide the trial registration number from ClinicalTrials.gov or an equivalent agency.*
Study protocol	*Note where the full trial protocol can be accessed OR if not available, explain why.*

Data collection	*Describe the settings and locales of data collection, noting the time periods of recruitment and data collection.*
Outcomes	*Describe how you pre-defined primary and secondary outcome measures and how you assessed these measures.*

Dual use research of concern

Policy information about dual use research of concern

Hazards

Could the accidental, deliberate or reckless misuse of agents or technologies generated in the work, or the application of information presented in the manuscript, pose a threat to:

No | Yes

- ☐ | ☐ Public health
- ☐ | ☐ National security
- ☐ | ☐ Crops and/or livestock
- ☐ | ☐ Ecosystems
- ☐ | ☐ Any other significant area

Experiments of concern

Does the work involve any of these experiments of concern:

No | Yes

- ☐ | ☐ Demonstrate how to render a vaccine ineffective
- ☐ | ☐ Confer resistance to therapeutically useful antibiotics or antiviral agents
- ☐ | ☐ Enhance the virulence of a pathogen or render a nonpathogen virulent
- ☐ | ☐ Increase transmissibility of a pathogen
- ☐ | ☐ Alter the host range of a pathogen
- ☐ | ☐ Enable evasion of diagnostic/detection modalities
- ☐ | ☐ Enable the weaponization of a biological agent or toxin
- ☐ | ☐ Any other potentially harmful combination of experiments and agents

Plants

Seed stocks	*Report on the source of all seed stocks or other plant material used. If applicable, state the seed stock centre and catalogue number. If plant specimens were collected from the field, describe the collection location, date and sampling procedures.*
Novel plant genotypes	*Describe the methods by which all novel plant genotypes were produced. This includes those generated by transgenic approaches, gene editing, chemical/radiation-based mutagenesis and hybridization. For transgenic lines, describe the transformation method, the number of independent lines analyzed and the generation upon which experiments were performed. For gene-edited lines, describe the editor used, the endogenous sequence targeted for editing, the targeting guide RNA sequence (if applicable) and how the editor was applied.*
Authentication	*Describe any authentication procedures for each seed stock used or novel genotype generated. Describe any experiments used to assess the effect of a mutation and, where applicable, how potential secondary effects (e.g. second site T-DNA insertions, mosiacism, off-target gene editing) were examined.*

ChIP-seq

Data deposition

☒ Confirm that both raw and final processed data have been deposited in a public database such as GEO.

☒ Confirm that you have deposited or provided access to graph files (e.g. BED files) for the called peaks.

Data access links<br>*May remain private before publication.*	https://www.ncbi.nlm.nih.gov/geo/query/acc.cgi?acc=GSE248866, reviewer access: opwpemisbyvrnuf
Files in database submission	Raw data in fastq.gz format, processed data: called peaks files in .narrowpeaks format, aligned genome tracks in .bigwig format.

Genome browser session
(e.g. UCSC)

Provide a link to an anonymized genome browser session for "Initial submission" and "Revised version" documents only, to enable peer review. Write "no longer applicable" for "Final submission" documents.

Methodology

Replicates	2 replicates per condition for each transcription factor.
Sequencing depth	40 million reads
Antibodies	8 µg of anti-GR (ProteinTech, #24050-1-AP) targets Glucocorticoid receptor<br>A cocktail of 4 µg of rabbit anti-FOXO1 (ProteinTech, #18592-1-AP)) and 4 µg of rabbit anti-FOXO1 (Abcam, #ab39670)
Peak calling parameters	The peaks were called with cutoff FDR = 0.01 and parameter set for BAM paired end.
Data quality	Number of peaks called with FDR 0.05 ranges from 12.000 to 40.000 for GR and from 7.500 to 13.000 for Foxo1. Meanwhile, under FDR 0.1 the ranges are from 13.000 to 48.000 for GR and from 8.900 to 14.000 for Foxo1. The FrIP score for each IPs range from 0.08 to 0.5 for FDR = 0.1.
Software	The quality of the reads was checked in FastQC ver. 0.11.9. Reads were aligned using BWA ver. 0.7.17, using the MEM algorithm79. The BAM files were sorted and indexed using Picard ver. 2.27.1. Duplicates and multimappers were removed using Picard and Bamtools ver. 2.5.2. Filtered BAM files were scored for flagstat using Samtools ver. 1.15.1 79. Peaks were called using MACS2 ver. 2.2.7.1. Peaks were filtered for blacklisted regions with bedtools version 2.30.0.Peak Universe: Peak universe table with unique genomic locations from all peaks by using bedtools Multiple Intersect in Galaxy ver. 2.30.0. We assumed as called peak in each group, if both replicates had a MACS2 peak and/or ranges with read count>50. The read count was generated by using featureCounts package. Bigwig tracks were generated from filtered BAM file using Deeptools ver. 3.5.1. We run peak annotation in Homer ver. 4.11. Heatmap and tag density plot was produced by using Deeptools ver. 3.5.1.

Flow Cytometry

Plots

Confirm that:

☐ The axis labels state the marker and fluorochrome used (e.g. CD4-FITC).

☐ The axis scales are clearly visible. Include numbers along axes only for bottom left plot of group (a 'group' is an analysis of identical markers).

☐ All plots are contour plots with outliers or pseudocolor plots.

☐ A numerical value for number of cells or percentage (with statistics) is provided.

Methodology

Sample preparation	*Describe the sample preparation, detailing the biological source of the cells and any tissue processing steps used.*
Instrument	*Identify the instrument used for data collection, specifying make and model number.*
Software	*Describe the software used to collect and analyze the flow cytometry data. For custom code that has been deposited into a community repository, provide accession details.*
Cell population abundance	*Describe the abundance of the relevant cell populations within post-sort fractions, providing details on the purity of the samples and how it was determined.*
Gating strategy	*Describe the gating strategy used for all relevant experiments, specifying the preliminary FSC/SSC gates of the starting cell population, indicating where boundaries between "positive" and "negative" staining cell populations are defined.*

☐ Tick this box to confirm that a figure exemplifying the gating strategy is provided in the Supplementary Information.

Magnetic resonance imaging

Experimental design

Design type	*Indicate task or resting state; event-related or block design.*
Design specifications	*Specify the number of blocks, trials or experimental units per session and/or subject, and specify the length of each trial or block (if trials are blocked) and interval between trials.*
Behavioral performance measures	*State number and/or type of variables recorded (e.g. correct button press, response time) and what statistics were used to establish that the subjects were performing the task as expected (e.g. mean, range, and/or standard deviation across subjects).*

Acquisition

Imaging type(s) — *Specify: functional, structural, diffusion, perfusion.*

Field strength — *Specify in Tesla*

Sequence & imaging parameters — *Specify the pulse sequence type (gradient echo, spin echo, etc.), imaging type (EPI, spiral, etc.), field of view, matrix size, slice thickness, orientation and TE/TR/flip angle.*

Area of acquisition — *State whether a whole brain scan was used OR define the area of acquisition, describing how the region was determined.*

Diffusion MRI ☐ Used ☐ Not used

Preprocessing

Preprocessing software — *Provide detail on software version and revision number and on specific parameters (model/functions, brain extraction, segmentation, smoothing kernel size, etc.).*

Normalization — *If data were normalized/standardized, describe the approach(es): specify linear or non-linear and define image types used for transformation OR indicate that data were not normalized and explain rationale for lack of normalization.*

Normalization template — *Describe the template used for normalization/transformation, specifying subject space or group standardized space (e.g. original Talairach, MNI305, ICBM152) OR indicate that the data were not normalized.*

Noise and artifact removal — *Describe your procedure(s) for artifact and structured noise removal, specifying motion parameters, tissue signals and physiological signals (heart rate, respiration).*

Volume censoring — *Define your software and/or method and criteria for volume censoring, and state the extent of such censoring.*

Statistical modeling & inference

Model type and settings — *Specify type (mass univariate, multivariate, RSA, predictive, etc.) and describe essential details of the model at the first and second levels (e.g. fixed, random or mixed effects; drift or auto-correlation).*

Effect(s) tested — *Define precise effect in terms of the task or stimulus conditions instead of psychological concepts and indicate whether ANOVA or factorial designs were used.*

Specify type of analysis: ☐ Whole brain ☐ ROI-based ☐ Both

Statistic type for inference — *Specify voxel-wise or cluster-wise and report all relevant parameters for cluster-wise methods.*

(See Eklund et al. 2016)

Correction — *Describe the type of correction and how it is obtained for multiple comparisons (e.g. FWE, FDR, permutation or Monte Carlo).*

Models & analysis

n/a	Involved in the study
☐	☐ Functional and/or effective connectivity
☐	☐ Graph analysis
☐	☐ Multivariate modeling or predictive analysis

Functional and/or effective connectivity — *Report the measures of dependence used and the model details (e.g. Pearson correlation, partial correlation, mutual information).*

Graph analysis — *Report the dependent variable and connectivity measure, specifying weighted graph or binarized graph, subject- or group-level, and the global and/or node summaries used (e.g. clustering coefficient, efficiency, etc.).*

Multivariate modeling and predictive analysis — *Specify independent variables, features extraction and dimension reduction, model, training and evaluation metrics.*
